# Prevention Starts in the Womb: Opportunities for Addressing Cardiovascular Risk Factors During Pregnancy and Beyond

**DOI:** 10.14797/mdcvj.696

**Published:** 2021-09-24

**Authors:** Lochan M. Shah, Alison Wand, Wendy Ying, Allison G. Hays, Roger S Blumenthal, Lili A Barouch, Sammy Zakaria, Garima Sharma

**Affiliations:** 1 Johns Hopkins University School Of Medicine, Baltimore, Maryland, US

**Keywords:** cardiovascular disease, prevention, pregnancy, post-partum, sex-specific risk factors, women’s health

## Abstract

Early identification and mitigation of sex-specific cardiovascular disease risk factors is a potential trajectory-changing strategy to improve lifelong cardiovascular health in women. These sex-specific risk factors include adverse pregnancy outcomes, polycystic ovarian syndrome, and premature menopause. We start by discussing the impact and management of risk factors for adverse pregnancy outcomes as an upstream intervention for cardiovascular disease risk reduction and then address the long-term effect and mitigation of sex-specific risk factors for cardiovascular disease.

## Introduction

Pregnancy is often described as “nature’s stress test” because it challenges maternal lipid, carbohydrate, and vascular homeostasis and can unmask underlying cardiovascular dysfunction. Thus, it offers a unique window into a woman’s future cardiovascular health and is an opportunity for early assessment and management of cardiovascular disease (CVD) risk factors. Traditional cardiovascular risk factors such as hypertension, dyslipidemia, and obesity increase the risk for adverse pregnancy outcomes (APOs),^1,2,3,4^ which include (1) preeclampsia (5% incidence in the United States^5^), (2) gestational hypertension/other hypertensive diseases of pregnancy (HDP) and gestational diabetes mellitus (GDM; 5.4% incidence^6^), (3) preterm birth (PTB; 10% incidence^7^), (4) small-for-gestational-age infant (SGA; 10% incidence^8^), (5) placental abruption (1% incidence^9^), (6) ischemic stroke (0.04% incidence^10^), and (7) maternal and neonatal mortality, among other complications of pregnancy. Conversely, APOs and other sex-specific diseases, such as polycystic ovarian syndrome (PCOS) and premature menopause,^11^ are also associated with higher risks of CVD.^12,13^ Therefore, upstream identification and mitigation of risk factors for APOs are also important. In this review, we discuss both the impact and management of risk factors for APOs and the long-term effects and mitigation of sex-specific risk factors for overall cardiovascular health.

## Impact and Management of Traditional CVD Risk Factors During Pregnancy

**Figure 1:**
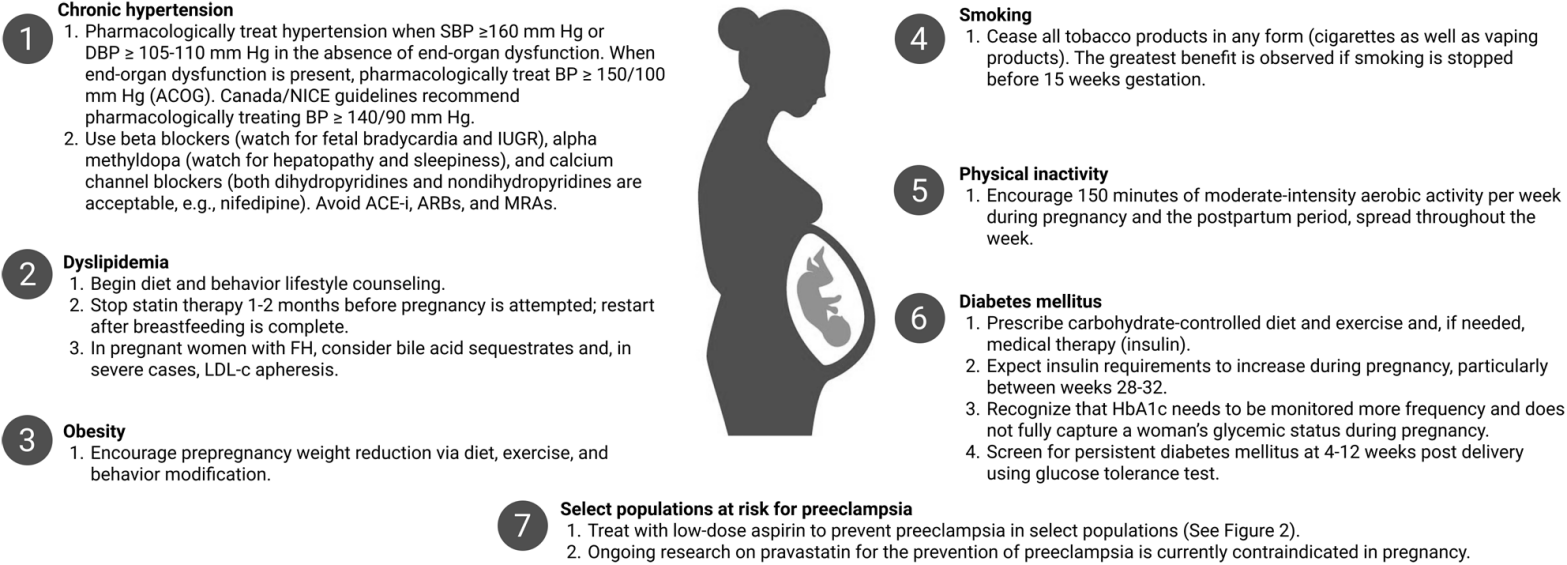
Management of adverse pregnancy outcome risk factors. SBP: systolic blood pressure; DBP: diastolic blood pressure; BP: blood pressure; ACOG: American College of Gynecologists; NICE: National Institute for Health and Care Excellence; ACE-i: angiotensin-converting enzyme inhibitor; ARBs: angiotensin receptor blockers; MRA: mineralocorticoid receptor antagonists FH: familial hypercholesterolemia; LDL-c: low-density lipoprotein cholesterol; HbA1c: hemoglobin A1c.

Cardiovascular disease is the primary cause of pregnancy-related mortality in the United States.^14^ This is partly due to the rising prevalence of traditional CVD risk factors including hypertension, obesity, and dyslipidemia.^15,16^ Worse pregnancy outcomes are especially pervasive in rural and racial minority communities. For example, the rate of prepregnancy hypertension has doubled in rural women in the past decade, and there are also higher rates of obesity, diabetes, and tobacco use in these vulnerable populations.^17,18,19^ Furthermore, many maternal risk factors also are associated with a higher relative risk of poor cardiovascular health in offspring during adolescence, potentially perpetuating poor cardiovascular health within these communities.^20^ Recognizing and managing these risk factors is a critical component of APO risk reduction and maternal and fetal cardiovascular care optimization (***Figure 1***).

### Chronic Hypertension

**Figure 2:**
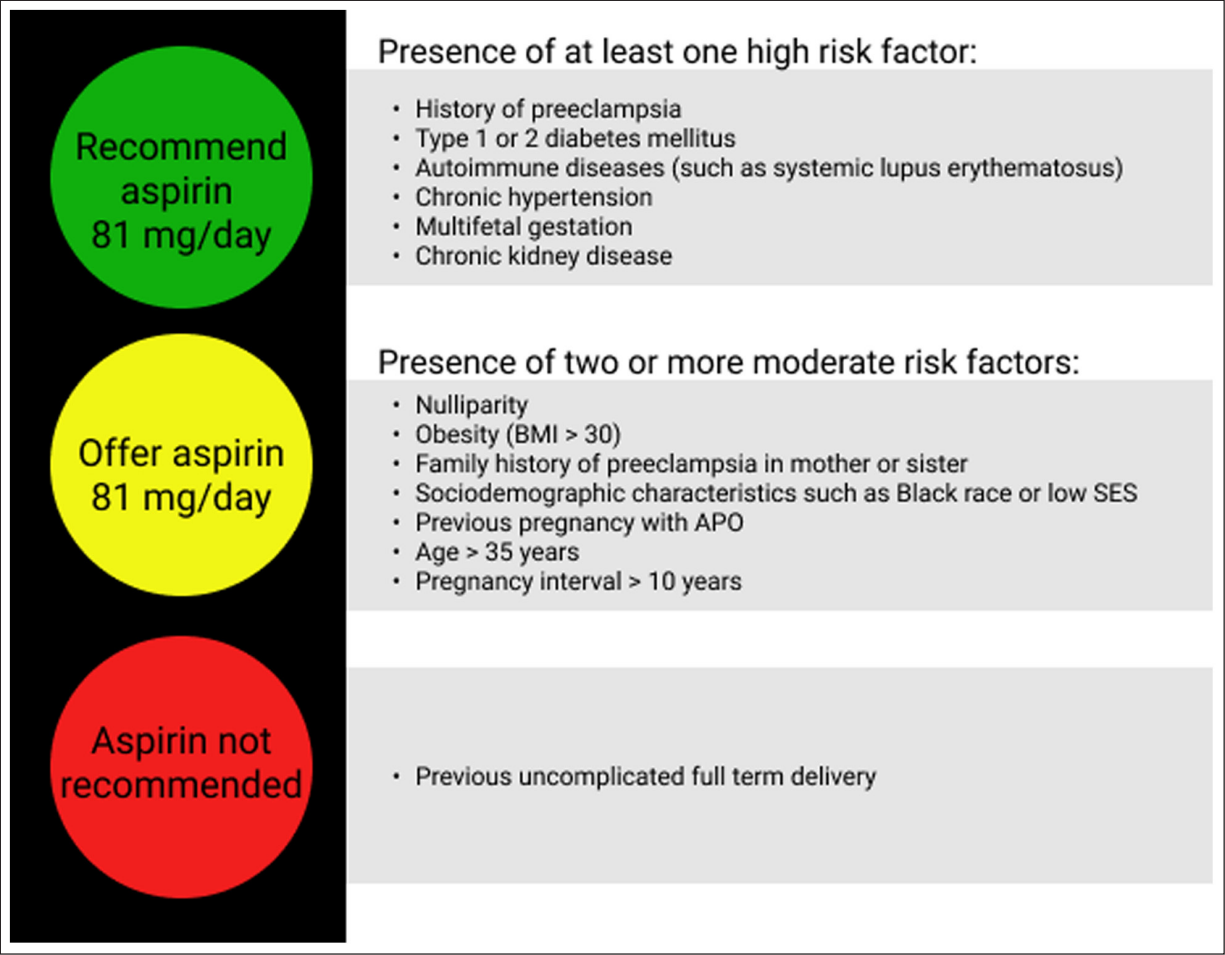
Aspirin use in the prevention of preeclampsia. SLE: systemic lupus erythematosus; BMI: body mass index; SES: socioeconomic status; APO: adverse pregnancy outcomes.

Chronic hypertension (CH) in pregnancy is defined by a systolic blood pressure (SBP) ≥ 140 mm Hg and/or diastolic blood pressure (DBP) ≥ 90 mm Hg before pregnancy or 20 weeks of gestation.^2^ Women with CH are at increased risk for APOs, particularly preeclampsia (***Figure 2***). A meta-analysis of 55 studies found relative risks (RR) of 7.7 for superimposed preeclampsia (95% CI, 5.7–10.1), 2.7 for PTB (95% CI, 1.9–3.6), and 1.3 for cesarean section (95% CI, 1.1–1.5).^21^ Women with CH also are more likely to experience pregnancies complicated by poor fetal growth (OR 3.0; 95% CI, 2.8–3.2)^1^ and placental abruption (RR 2.4; 95% CI, 2.3–2.5).^2^ Notably, women with CH who develop preeclampsia are at even higher risk for other APOs and often develop preeclampsia earlier than those without CH.^2^ Finally, women with CH are at higher risk of cerebrovascular complications (OR 5.4; 95% CI, 4.3–6.9).^1^

## Management of Chronic Hypertension During Pregnancy

Although the American College of Cardiology (ACC) and the American Heart Association (AHA) have well-established guidelines for the management of chronic hypertension in nonpregnant individuals, optimal goal ranges for blood pressure management during pregnancy are highly debated. Overly aggressive blood pressure control may decrease fetoplacental perfusion.^2^ A 2011 Cochrane review of two studies including 256 women found that tight blood pressure control was associated with a higher risk of hospital admission (RR 2.53; 95% CI, 1.14–5.63) without differences in severe preeclampsia or fetal growth restriction.^22^ A subsequent Cochrane review of 31 trials including 3,485 women with mild-to-moderate hypertension during pregnancy (SBP 140–169 mm Hg and DBP 90–109 mm Hg) found that treatment reduced risk of severe maternal hypertension—defined as SBP ≥ 170 mm Hg and/or DBP ≥ 110 mm Hg on two consecutive occasions 15 minutes apart (RR 0.49 in 20 trials; 95% CI,0.40–0.60)—but did not significantly reduce the risk of APOs (including preeclampsia, fetal/neonatal death/miscarriage, SGA, and PTB).^23^

On the other hand, the Control of Hypertension in Pregnancy Study (CHIPS) randomized women with CH or gestational hypertension to “tight” or “less tight” control (target DBP 85 vs 100 mm Hg, respectively) and found that “tight control” was protective against severe maternal hypertension; in addition, a post-hoc analysis found that severe hypertension was a risk marker for APOs, independent of blood pressure control or preeclampsia.^24^ Post-hoc analysis also found that “less tight” blood pressure control appeared to result in a lower incidence of SGA babies (OR 0.66; 95% CI, 0.44–1.00).^25^

Based on these studies, the American College of Obstetrics and Gynecology (ACOG) recommends pharmacological treatment only for severe hypertension in pregnancy (SBP ≥ 160 mm Hg or DBP ≥ 105–110 mm Hg) in the absence of end-organ dysfunction. In the presence of end-organ dysfunction, antihypertensives are recommended at a blood pressure ≥ 150/100 mm Hg. Additionally, ACOG recommends discontinuing antihypertensives in women with mild CH (140-160/90-110 mm Hg) during pregnancy.^26^ In contrast, influenced by the CHIPS data, Canada and UK guidelines both recommend treating blood pressure ≥ 140/90 mm Hg in pregnant women with hypertension.^27,28^ The ongoing CHAP trial (Chronic Hypertension and Pregnancy Project) hopes to address this question more definitively.^25^

Recommended antihypertensive medications during pregnancy include beta blockers, alpha methyldopa, and calcium channel blockers.^29^ Labetalol is the preferred beta blocker, although metoprolol is an acceptable alternative.^30^ When using beta blockers, careful fetal monitoring is advised because of associated fetal bradycardia and intrauterine growth restriction.^29,31^ For calcium channel blockers, both dihydropyridines and nondihydropyridines are acceptable, though nifedipine is most commonly used.^29,32^ Angiotensin converting enzyme inhibitors, angiotensin receptor blockers, and mineralocorticoid receptor antagonists are strongly contraindicated during pregnancy due to risk for fetal abnormalities.^29,31^

### Dyslipidemia

Triglycerides, total cholesterol, and low-density lipoprotein-c (LDL-C) increase during pregnancy and drop following delivery.^33,34^ Physiologic changes in total cholesterol and triglycerides are thought to be nonatherogenic. However, pregnancy is also associated with an increased proportion of smaller, denser LDL-C particles that may be more atherogenic, with higher concentrations correlating with an increased risk of CVD later in life.^34^ Elevated levels of lipids in predisposed women carry increased risk for APOs.^33,34^

The Amsterdam Born Children and their Development cohort study discovered a linear association between elevated maternal triglyceride levels in early pregnancy and pregnancy-induced hypertension (OR 1.6; 95% CI, 1.1–2.3), preeclampsia (OR 1.69; 95% CI, 1.1–2.6), and induced PTB (OR 1.69; 95% CI, 1.2–2.5).^4^ Additionally, the Coronary Artery Risk Development in Young Adults study found an independent correlation between prepregnancy total cholesterol levels that were either low (< 156 mg/dL) or high (> 195 mg/dL) with PTB. Based on these results, low prepregnancy cholesterol may be a marker of poor nutritional status or a baseline inflammatory state that leads to PTB-inducing cascade, whereas high prepregnancy cholesterol represents a “proatherogenic phenotype” with increased risk of PTB.^35^

## Management of Dyslipidemia During Pregnancy

Behavioral and lifestyle counseling are the cornerstones of management for dyslipidemia in pregnancy.^34,36,37^ Drug therapy is limited because of concerns for teratogenicity, with current guidelines recommending against the use of many drugs during pregnancy and lactation.^33,34,36,37^ In particular, the 2018 AHA Cholesterol Guidelines recommend stopping statin therapy 1 to 2 months before conception and restarting after cessation of breastfeeding.^37^ Despite these recommendations, ongoing trials are evaluating pravastatin in pregnant women to prevent preeclampsia, because hydrophilic statins such as pravastatin or rosuvastatin are potentially less associated with fetal malformations than lipophilic statins such as simvastatin or atorvastatin.^37,38^

Other medications, including ezetimibe, niacin, and fibrates, are similarly associated with teratogenicity and are thus contraindicated during pregnancy.^33^ Proprotein convertase subtilisin/kexin type 9 serine protease inhibitors have not been tested for safety during pregnancy and are not currently approved during pregnancy.^33^ Safe therapies are limited to bile acid sequestrants and LDL-C apheresis, both of which are only approved in women with severe familial hypercholesterolemia.^33,39^

### Obesity

There is growing evidence linking obesity (body mass index [BMI] ≥ 30 kg/m^2^) with a multitude of APOs.^3^ A subgroup of women with obesity enrolled in the Cardiac Disease in Pregnancy (CARPREG II) study had a higher risk of experiencing a cardiac event (OR 1.7; 95% CI, 1.0–2.7) or preeclampsia (8% vs. 2%; *P* = .001) compared with women of normal weight.^40^ Obese women were more likely to present with heart failure in pregnancy (8% vs 3%; *P* = .02) compared with normal-weight women (BMI 18.5–24.9 kg/m^2^). Risk of maternal mortality also has been linked to increasing BMI. A population-based case-control study in France found that overweight women (BMI 25–29.9 kg/m^2^) had an OR of 1.7 (95% CI, 1.3–2.2) for maternal death, and those with class 2 to 3 obesity (BMI ≥ 35 kg/m^2^) had an OR of 3.4 (95% CI, 2.2–5.3), compared with those who have normal BMI.^41^

Obesity was also associated with an increased risk of fetal complications such as PTB (19% vs 10%; *P* = .05) and respiratory distress syndrome (8% vs. 3; *P* = .02).^40^ Women who were overweight but not obese also had higher rates of infant mortality than women with normal weight.^42^ Finally, maternal obesity portends long-term risks for offspring, including an increased risk of metabolic syndrome and childhood obesity, even after adjustment for complications such as GDM.^3^

## Management of Obesity in Pregnancy

To mitigate obesity-related complications of pregnancy, clinical guidelines from ACOG and the National Institute for Health and Care Excellence recommend weight-related screening and preconception care.^3^ Obese women who have even small weight reductions before pregnancy have improved pregnancy outcomes.^3^ Overweight and obese women should also receive counseling on the increased risks of pregnancy complications and should be encouraged to engage in prepregnancy weight reduction via diet, exercise, and behavior modification.^3,43^

### Physical Inactivity

In pregnancy, physical inactivity is an independent risk factor for maternal obesity and related pregnancy complications, including GDM.^44,45^ Moreover, aerobic exercise has been associated with positive pregnancy outcomes such as less gestational weight gain and lower incidences of GDM, gestational hypertension, PTB, and cesarean delivery.^45,46^ Pregnant women should continue to or initiate safe physical exercise in the absence of obstetric or medical contraindications.

The US Department of Health and Human Services Physical Activity Guidelines recommend at least 150 minutes of moderate-intensity aerobic activity per week during pregnancy and the postpartum period, spread throughout the week.^47^ Examples of such aerobic activities include walking, stationary cycling, dancing, water aerobics, and use of resistance exercises.^45,46^

### Smoking

Cigarette smoking is associated with many APOs.^48^ Active smoking is associated with increased risk of miscarriage (RR 1.23; 95% CI, 1.2–1.3)^49^ and stillbirth (RR 1.5; 95% CI, 1.4–1.5).^50^ Maternal smoking also has a dose-dependent relationship with obesity and short stature in adult daughters; in fact, 61% of offspring of heavier smokers (≥ 10 cigarettes/day) and 37% of light smokers (< 10 cigarettes/day) are more likely to be obese than the daughters of nonsmokers.^51^ It should be noted that harmful effects on pregnancy also occur with the use of other nicotine products, including vaping products with nicotine or nicotine salts.^48^ Smoking cessation at any point in gestation is beneficial to the pregnant woman and her fetus, although the greatest benefit occurs with smoking cessation prior to 15 weeks of gestation.^48^ Thus, cessation of all tobacco products should be strongly encouraged as early as possible.

To accomplish this, the most recent US Preventive Services Task Force (USPSTF) Guidelines recommend behavioral interventions because current evidence was determined to be insufficient to assess the balance of benefits and harms of pharmacotherapy (eg, nicotine replacement therapy) for tobacco cessation in pregnant women.^52^ Trials that do exist for nicotine replacement therapy show inconsistent data,^48,52^ with most trending towards benefit, although some trials were terminated early due to concerns from data/safety monitoring committees.^48^ The USPSTF also identified no strong evidence base to support varenicline and bupropion, other commonly used pharmacologic agents in nonpregnant patients, during pregnancy.^52^

### Diabetes Mellitus

Pregnancy in healthy women is associated with a component of insulin resistance, which may be needed to deliver adequate nutrition to the developing fetus. Insulin resistance rises throughout pregnancy, peaks in the third trimester, and returns to prepregnancy levels after delivery.^53^ However, frank diabetes (or prediabetes) is deleterious to the fetus and associated with APOs.^54,55^ Women who develop GDM are almost twice as likely to develop postpartum diabetes.^55,56^ Pregnant women with preconception type 1 or type 2 diabetes have become more prevalent in recent years^54^ and have a significantly higher risk of preeclampsia^53,57^ and acute myocardial infarction during pregnancy.^58^

The management of diabetes in pregnancy includes a carbohydrate-controlled diet, exercise, and medical therapy to achieve euglycemia.^59^ Insulin is the preferred medical treatment for patients who are not adequately controlled by diet and exercise, and providers should expect insulin requirements to increase during pregnancy, particularly between 28 and 32 weeks gestation.^55^ Given the increased red blood cell turnover and the physiologic changes of pregnancy, HbA1c needs to be monitored more frequently, and even then it may not fully capture a woman’s glycemic status during this time.^55,59^ Finally, women with GDM should be tested for persistent diabetes mellitus for 4 to 12 weeks after delivery using an oral glucose tolerance test.^59^

### Additional Strategies to Mitigate Risk Factors During Pregnancy

Low-dose aspirin reduces the risk of APOs in women who are at increased risk for preeclampsia. A 2019 Cochrane Review of 77 trials found an 18% reduction in proteinuric preeclampsia (RR 0.82; 95% CI, 0.77–0.88), fetal or neonatal death (RR 0.85; 95% CI, 0.76–0.95; 14% risk reduction), PTB (RR 0.91; 95% CI, 0.87–0.95; 9% risk reduction), and SGA babies (RR 0.84; 95% CI, 0.76–0.92).^60^ However, antiplatelet agents made little or no difference in the risk of HELLP syndrome, severe maternal morbidity, or admission to a newborn special care unit.^60^ Similarly, the USPSTF performed a systematic review and found that low-dose aspirin reduced the risk for preeclampsia by 24%, PTB by 14%, and intrauterine growth restriction by 20%.^61^

Thus, both USPSTF and ACOG recommend the prophylactic use of low-dose aspirin (81 mg/d) between 12 and 16 weeks gestation in women with the preeclampsia risk factors noted in ***Figure 1***.^61^ In addition to optimal control of the above risk factors, aspirin in at-risk women and prepregnancy weight loss in obese and overweight women, there are also several therapies under investigation, including statins. Although they are classified as pregnancy category X by the FDA,^62^ several prospective human studies (NCT03648970, NCT01717586) are investigating the role of pravastatin in preeclampsia prevention and treatment and should have results in the next few years.^63,64^

## Long-Term Effect of Sex-Specific Risk Factors on Cardiovascular Health

### Adverse Pregnancy Outcomes

**Figure 3:**
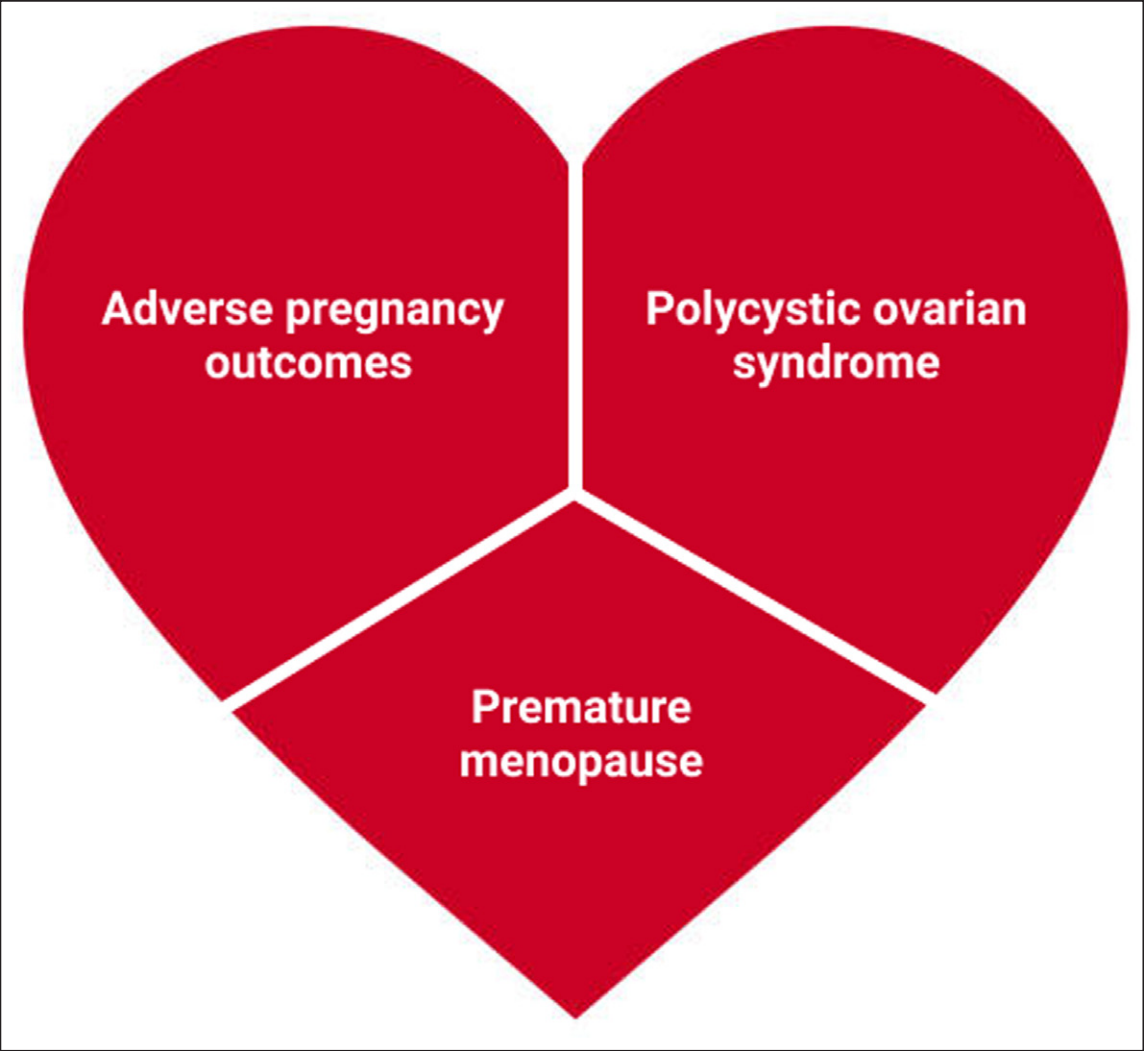
Sex-specific cardiovascular risk factors in women.

Adverse pregnancy outcomes result in short-term complications during pregnancy and are strongly associated with an increased long-term risk for CVD such as heart failure, coronary artery disease, and stroke (***Figure 3***).^12^ In particular, preeclampsia predicts increased CVD risk before the emergence of traditional CVD risk factors.^65^ Compared with women with uncomplicated pregnancy, women who have had preeclampsia have a four-fold increased risk of heart failure and a two-fold increased risk of coronary artery disease, heart failure, stroke, and CVD death.^66^ Preeclampsia and preterm birth are also associated with an increased risk of CVD-related death, with OR 1.73 (95% CI, 1.46–2.06) and 1.93 (95% CI, 1.83–2.03) respectively.^12^ Women with gestational hypertension or GDM are 67% more likely to have subsequent CVD compared to women without these adverse pregnancy outcomes.^12^ In addition, women who have had HDP are more likely to develop chronic hypertension after pregnancy.^67^ Finally, pregnancy loss is also associated with greater odds of future CVD even after adjustment for CVD risk factors.^68^ For these reasons, APOs such as preeclampsia and preterm delivery are now considered risk-enhancing factors in the assessment of atherosclerotic risk to guide statin therapy for asymptomatic women at intermediate risk of atherosclerotic cardiovascular disease.^69^

### Polycystic Ovarian Syndrome

PCOS presents during a woman’s reproductive years and contributes to subfertility, APOs, and potentially higher rates of CVD.^70,71,72^ PCOS is associated with a higher prevalence of central adiposity, insulin resistance, dyslipidemia, hypertension, obstructive sleep apnea, metabolic syndrome, and obstructive sleep apnea.^73,74,75^ In pregnancy, women with PCOS have higher rates of GDM (OR 2.8; 95% CI, 1.9–4.1) and HDP (OR 4.1; 95% CI, 2.8–6.0).^70^ Notably, PCOS appears to be an independent risk factor for GDM and preeclampsia even after controlling for obesity, multiple pregnancy, and low parity.^74^

Women with PCOS have a higher prevalence of subclinical CVD as measured by coronary artery calcium, carotid intima-media thickness, and endothelial function via flow-mediated brachial artery dilatation.^72^ However, the degree of CVD in women with PCOS is unclear, and studies have demonstrated mixed findings. A recent study of 60,574 women with PCOS in the Danish Registry Cohort found a 19% higher risk of developing CVD compared with women who did not have PCOS when adjusted for age and obstetric and social risk factors.^76^

However, when stratified by age, women with PCOS aged 50 and older did not have a higher cardiovascular risk, while women in their 30s and 40s with PCOS trended towards being at greater risk of CVD compared with those without PCOS. The diminishing difference in older women may be due to the confounding positive association between age and CVD risk factors.^76^ Accounting for this confounding, other studies have suggested that the absolute risk conferred by PCOS is negligible, although certain phenotypes of women with PCOS are at higher risk.^71^ More well-designed trials are needed to assess the true risk of CVD due to PCOS and guide risk stratification. Until then, we advocate that PCOS be treated as a significant risk factor for CVD regardless of BMI, with aggressive treatment of comorbid conditions such as diabetes and hyperlipidemia.

### Premature Menopause

The postmenopausal state is a well-established risk factor for CVD.^77^ Premature menopause and primary ovarian failure, now termed primary ovarian insufficiency (POI), is the depletion or dysfunction of ovarian follicles with cessation of menses before age 40.^78^ Similarly, women with the cessation of menstruation before age 45 are considered to have early-onset menopause. Early loss of residual ovarian follicles and consequent estrogen deficiency results in a cascade of negative downstream cardiovascular effects.^78^ In particular, estrogen deficiency is associated with less-compliant blood vessels and endothelial dysfunction due to activation of the renin-angiotensin-aldosterone system.^77^ Estrogen deficiency also leads to adverse lipid metabolism and atherosclerotic plaque development.^78^

Unsurprisingly, women with POI have an increased risk of CVD and mortality.^77,78^ A 2020 cohort study of 144,260 postmenopausal women in the UK found that menopause before age 40 was associated with increased risk (HR 1.4; 95% CI, 1.2–1.6) for a composite outcome that included CAD, heart failure, aortic stenosis, mitral regurgitation, atrial fibrillation, ischemic stroke, peripheral artery disease, and venous thromboembolism after adjustment for conventional CVD risk factors and the use of menopausal hormone therapy.^79^ A meta-analysis of 32 observational studies found that women with premature menopause (age < 40 years) had an increased risk of CAD (RR 1.5; 95% CI, 1.3–1.8).^77^ In addition, women who underwent menopause between ages 50 to 54 years versus at age 50 and younger had a decreased risk of fatal coronary heart disease (RR 0.87; 95% CI, 0.80–0.96). A 2019 pooled analysis of individual patient data from 15 observational studies found that women with premature and early menopause (age < 40 vs < 45, respectively) had a significantly increased risk of a nonfatal CVD event before age 60, with HR 1.6 (95% CI, 1.4–1.7) and 1.3 (95% CI, 1.2–1.4), respectively, compared to women with natural menopause (age 50–51).^80^

## Management of Premature Menopause

Based on these and other data, recent updates to the ACC/AHA cholesterol guidelines include premature menopause (prior to age 40) as a risk enhancer and warrant stronger consideration for statin therapy.^37^ In addition, unless there is an absolute contraindication to taking estrogen therapy, ACOG recommends hormone therapy in women with POI until the average age of natural menopause (age 50–51).^78^ In women with POI, hormone therapy improves endothelial dysfunction and blood pressure, although the results from the Women’s Health Initiative trials related to menopause therapy are not fully applicable to women with POI in terms of cardiovascular risk-to-benefit assessment.^78^ More studies are needed to assess the direct impact of hormone therapy in women with POI on incident cardiovascular events. At the time of writing, evidence is insufficient to support hormone therapy for the sole purpose of preventing CVD in the absence of premature menopause.

## Conclusion

Cardiovascular disease remains the leading cause of death for women, necessitating early and active intervention. The peripartum period has the potential to serve as a catalyst for behavior change because women may be more receptive to making lifestyle changes before, after, and during pregnancy.^65,64^ Recognizing and mitigating risk factors for APOs has the added benefit of also improving risk-factor profiles for long-term CVD and should be a goal of care. Finally, screening for both traditional and sex-specific cardiovascular risk factors by all providers—primary care physicians, obstetricians/gynecologists, and cardiologists—offers an upstream, potentially trajectory-changing strategy to optimize long-term cardiovascular health in women.

## Key Points

- In addition to screening for traditional cardiovascular risk factors, all providers caring for women should screen for sex-specific risk factors such as adverse pregnancy outcomes, polycystic ovarian syndrome, and premature menopause.
- Traditional cardiovascular risk factors such as chronic hypertension, dyslipidemia, obesity, smoking, physical inactivity, and diabetes increase the risk for adverse pregnancy outcomes.
- Risk from sex-specific cardiovascular risk factors can be mitigated with early detection and intervention.
- Pregnancy is an opportune time to identify women who are at risk for future cardiovascular disease, intervene early, and not only improve pregnancy outcomes but also improve long-term cardiovascular health.
